# Aquaculture Enclosures Relate to the Establishment of Feral Populations of Introduced Species

**DOI:** 10.1371/journal.pone.0006199

**Published:** 2009-07-13

**Authors:** Xuan Liu, Yiming Li

**Affiliations:** 1 Key Laboratory of Animal Ecology and Conservation Biology, Institute of Zoology, Chinese Academy of Sciences, Chaoyang, Beijing, China; 2 Graduate University of Chinese Academy of Sciences, Shijingshan, Beijing, China; Trinity College Dublin, Ireland

## Abstract

Many species introduced by humans for social and economic benefits have invaded new ranges by escaping from captivity. Such invasive species can negatively affect biodiversity and economies. Understanding the factors that relate to the establishment of feral populations of introduced species is therefore of great importance for managing introduced species. The American Bullfrog (*Lithobates catesbeianus*) is one species that has escaped from farms, and it is now found in the wild in China. In this study, we examined influences of two types of bullfrog farm (termed simple and elaborate farm enclosures) on the establishment of feral populations of this species in 137 water bodies in 66 plots in four provinces of China. The likelihood of establishment of bullfrog populations in water bodies in plots with simple enclosures (49/89 = 55.1%) was higher than those with elaborate enclosures (3/48 = 6.3%). Based on the Akaike Information Criterion, the minimum adequate model of generalized linear mixed models with a binomial error structure and a logit link function showed that the establishment or failure of bullfrog populations in water bodies was positively correlated with the presence of a simple enclosure, the number of bullfrogs raised and the presence of permanent water in a plot, but negatively correlated with distance from a bullfrog farm and the occurrence of frequent hunting. Results therefore suggest that a simple farm enclosure can increase the establishment of feral bullfrog populations compared with an elaborate enclosure. Our findings are the first to quantify the importance of improving farming enclosures to control and minimize the risk from introduced species.

## Introduction

Many species introduced for social and economic benefits [1] often invade new areas after escaping from holding enclosures. Such invasive species can have a severe effect on native species, communities, ecosystems and economies [2]–[4]. The development of effective measures to cope with species invasions is urgently needed [5]. Invasions are a complex process comprised of three main steps: initial escape, establishment of feral populations, and spread into the recipient habitat [6], [7]. Once they are established, the eradication of an introduced species is extremely costly and difficult [8], [9]. Therefore, preventing the establishment of feral populations of an alien species has been widely accepted as one of the most promising and cost-effective management strategies [10], [11]. Understanding the factors related to the establishment of feral populations of introduced species is therefore of great importance for preventing any invasion [12], [13].

Although many factors, such as invader intrinsic traits [14], climate/habitat match [15], species interactions [16], transportation pathways [17], human affiliations [18] and socioeconomic factors [19] can influence the establishment of feral populations of invasive species, propagule pressure has been regarded as a key establishment indicator [20]. Propagule pressure refers to the estimate of the absolute number of individuals involved in any one release event and the number of discrete release events [21]. It increases with an increasing number of released individuals and the number of release events, thus promoting the likelihood of establishment.. Different factors can affect the number of released individuals and number of release events in the establishment of feral populations of introduced species. For example, the volume and frequency of transporting introduced species, and different operations in transport pathways, can affect the supply of propagules and thereby invasion risk [22]. Once the introduced species arrive at a new site for captive breeding, the number of introduced individuals, and farming practices can affect propagule pressure [23]–[26]. Increasing the number of introduced individuals and frequency of cultivation can increase the likelihood of establishment of feral populations. The quality of farming enclosures can also affect the establishment or failure of feral populations of introduced plants and animals. All else being equal, good quality enclosures may reduce the escape of individuals and therefore reduce the likelihood of the establishment. Although it is widely accepted that a poorly constructed enclosure leads to an increase in escapees [27], there are few quantitative estimates of the influence of different farming enclosures on the likelihood of the establishment of introduced species [28], [29].

We examined the influence of American bullfrog (*Lithobates catesbeianus*; hereafter referred to as the bullfrog) aquaculture enclosures on the establishment of feral populations of this species in different water bodies in Hubei, Hunan, Guizhou and Yunnan provinces of China. The bullfrog is listed as one of the 100 worst invasive alien species in the world [30]. Native to eastern North America, bullfrogs have been introduced into many countries, and they appear to have caused the decline or extinction of some native amphibians [31]–[36]. Bullfrogs also often co-occur with other aquatic invasive species [33], [37], [38]. The invasion of bullfrogs can be facilitated by the presence of co-evolved non-native fish [39]. Bullfrogs were introduced into mainland China in 1959 and they have been raised for human consumption on farms in most provinces since then [40]. Populations of bullfrogs that have escaped from farms have recently invaded native frog communities in many natural water bodies. Captive bullfrogs are generally raised in one of two types of farm enclosure: a simple enclosure or an elaborate enclosure. The latter is a building constructed of brick and concrete within which is a man-made pond for breeding bullfrogs, or a shed constructed of plastic film that tightly covers a natural pond. This enclosure is more expensive, but is more effective in preventing bullfrog escape. The simple enclosure is a low cost enclosure built in rice fields, pools or reservoirs on farmers' property. Fences are 1.5 meter high and made of fragile fiberglass tiles or polyethylene mesh, which are easily destroyed by storms and heavy rains. The majority of farmers raise bullfrogs in simple enclosures. We predicted that such a farm enclosure would increase the likelihood of establishment of feral bullfrog populations because they would be able to escape more easily from it.

We investigated and compared the establishment or failure of feral bullfrog populations in water bodies in circular plots (a plot had radius of 1000 m) centered within the two types of bullfrog farm, as well as the characteristics of the farms, their water bodies, and human activities in Hubei, Hunan, Guizhou and Yunnan provinces of China. In addition, we determined factors that relate to the establishment of feral bullfrog populations in water bodies by linking the establishment or failure to characteristics of the bullfrog farms, the water, and human activities around the water bodies.

## Results

**Figure 1:**
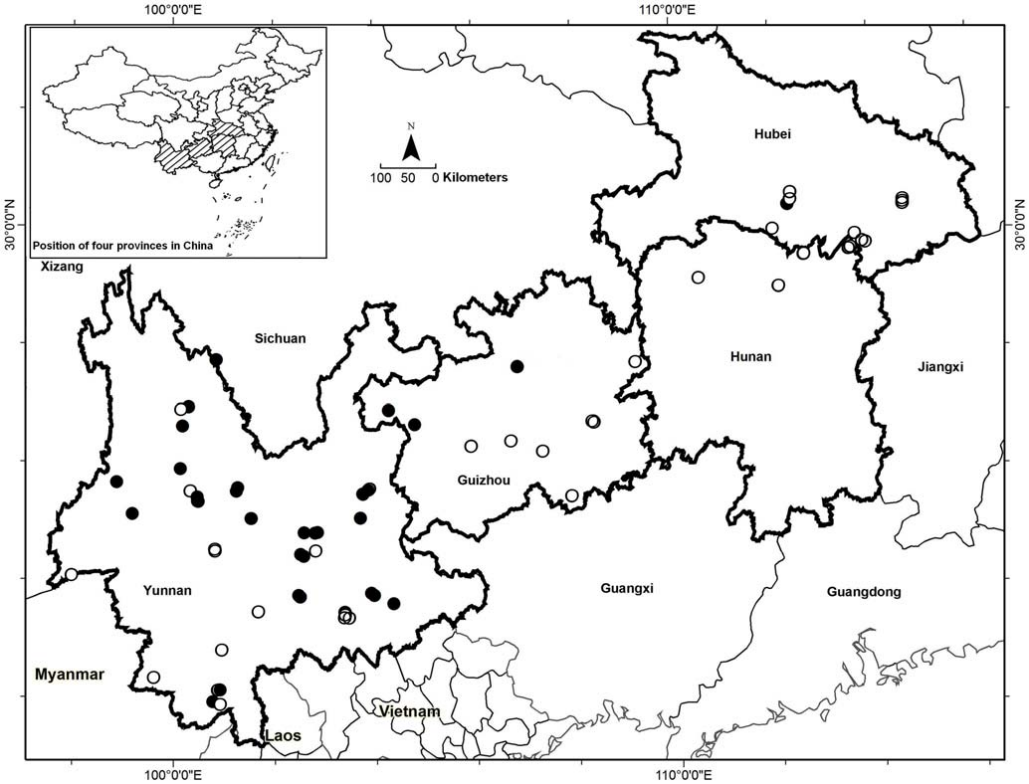
Distribution map of *L. catesbeianus* in the study area. Filled circles show water bodies with bullfrog population establishment and open circles without bullfrog population establishment. Some points are superimposed.

We investigated 137 water bodies in 66 plots: 52 water bodies in 15 plots in Hunan and Hubei provinces, 27 water bodies in nine plots in Guizhou province and 56 water bodies in 42 plots in Yunnan province. Bullfrog populations had become established in 52 water bodies in 38 plots in all four provinces: six water bodies in five plots in Hunan and Hubei, four water bodies in three plots in Guizhou, and 42 water bodies in 30 plots in Yunnan (Figure 1). Of 66 plots, 50 (89 water bodies) contained a bullfrog farm with a simple enclosure, and 16 (48 water bodies) had an elaborate enclosure. The water bodies in plots that contained a farm with a simple enclosure (49/89 = 55.1%) were more likely to have bullfrog populations than those at an elaborate enclosure (3/48 = 6.3%, see Table 1). These two types of farms were also significantly different in several other respects (Table 1). Farms with a simple enclosure tended to start earlier and have raised bullfrogs for a shorter duration when compared with those with an elaborate enclosure. Furthermore there was a difference in altitude and climate variables between plots with a simple enclosure and those with an elaborate enclosure.

**Table 1:** Comparison of bullfrog population establishment and predictor variables in water bodies (n = 137) between plots with a simple enclosure and those with an elaborate enclosure in Hunan, Hubei, Guizhou and Yunnan Provinces, China. Values are proportions or means (±SE).

Predictor variables	Simple enclosure	Elaborate enclosure	Test a
Proportion of bullfrog population establishment	49/89	3/48	29.5***
Log_10_ (number of bullfrogs raised)	3.5±0.10	3.35±0.06	1.6
Duration (years)	3.5±0.11	4.2±0.16	3.3*
Time since a farm raised bullfrogs (years)	11.4±0.52	9.5±0.48	2.7*
Proportion of frequent Hunting	55/89	27/48	0.2
Occurrence of a permanent water body	62/89	40/48	2.4
Water body max. depth (m)	2.0±0.10	2.3±0.18	1.4
Vegetation cover (category)^b^	3.3±0.11	3.3±0.22	0.2
Log_10_ (area of water body) (m^2^)	3.4±0.11	3.3±0.20	0.6
Shading	23.3±1.35	21.0±1.42	1.1
Occurrence of red swamp crayfish	48/89	33/48	2.3
Occurrence of nonnative fish	46/89	19/48	1.4
Species richness of native frog communities	4.4±0.06	4.4±0.13	0.2
Distance to the farm (km)	0.4±0.03	0.3±0.02	1.8
Human footprint	37.2±1.47	34.4±2.47	1.1
Altitude (m)	1143.4±82.24	368.27±77.95	6.8***
Tmax (°C)	29.0±0.36	31.48±0.28	5.4***
Tmin (°C)	3.3±0.33	1.7±0.24	4.1***
Prec0608 (mm)	524.4±8.06	501.5±12.26	1.6
Prec1202 (mm)	71.8±4.68	111.2±5.31	5.3***

* *P*<0.05.

*** *P*<0.001.

a t-values from a comparison of the means for continuous variables, and χ^2^-values from a test of independence for categorical variables.

b Category is the unit for vegetation cover (see text).

The mixed models with a binomial error structure and a logit link function for a single variable showed that the probability of establishment of a bullfrog population in a water body was positively correlated to the occurrence of simple enclosures (*β* = 4.53, *p* = 0.006), the number of bullfrogs raised (*β* = 5.44, *p*<0.001), permanent water (*β* = 4.14, *p* = 0.004), time since a farm raised bullfrogs (*β* = 0.47, *p* = 0.009), human footprint (*β* = 0.09, *p*<0.001) and altitude (*β* = 0.002, *p*<0.001). However, the likilihood of establishment of bullfrog populations was negatively correlated to frequent hunting (*β* = −8.18, *p*<0.001), the distance of a water body to a farm in a plot (*β* = −30.24, *p*<0.001), Tmax (maximum temperature of the hottest month) (*β* = −0.61, *p*<0.001) and Prec1202 (summed precipitation between December and February) (*β* = −0.04, *p*<0.001).

We used multivariate mixed models and the Akaike Information Criterion to determine a minimum adequate model (MAM). A full model contained the establishment or failure of bullfrog population as a responsible variable and the number of bullfrogs raised, the occurrence of frequent hunting, the human footprint, distance to a farm, the occurrence of permanent water, time since a farm raised bullfrogs, altitude, Tmax (maximum temperature of the hottest month), Tmin (minimum temperature of the coldest month), Prec0608 (summed precipitation between June and August), Prec1202 (summed precipitation between December and February), and the occurrence of a simple enclosure in a plot as independent variables. The MAM included the occurrence of a simple enclosure, permanent water, distance to a farm, number of bullfrogs raised, and the occurrence of frequent hunting (Table 2). These indicated that a water body was more likely to have a feral bullfrog population if farmers raised them using a simple enclosure, raised more bullfrogs in a plot, hunting was infrequent, or if the water body was permanent and closer to a farm.

**Table 2:** Results of the minimum adequate model based on generalized linear mixed models with the population establishment of *L. catesbeianus* to the explanatory variables (n = 137). Non-significant variables are not reported. The multivariate model was the best model according to the Akaike's Information Criterion (AIC). *β*:regression coefficient; *P*: likelihood Ratio Test. The AIC for the minimum adequate model was 66.32.

Explanatory variables	*β*	SE	*P*
Presence of simple enclosure	4.53	0.97	<0.001
Presence of permanent water bodies	2.91	1.60	0.069
Occurrence of frequent hunting	−2.04	0.86	0.018
Log_10_ (number of bullfrogs raised)	3.10	0.96	0.001
Distance to farm	−6.63	2.09	0.002

## Discussion

This study provided quantitative evidence for the role of bullfrog farms in introducing this species into the wild in China. The likelihood of bullfrog populations becoming established was higher in water bodies of plots with a simple enclosure than those with an elaborate one, and was positively correlated with the occurrence of a simple enclosure in plots. These results confirmed our prediction that a simple farm enclosure would increase the likelihood of establishment of feral bullfrog populations in comparison to an elaborate enclosure. There were no differences in the variables (occurrence of permanent water, number of bullfrogs raised, occurrence of frequent hunting, and distance to a farm) retained in the MAM between plots with a simple enclosure and those with an elaborate one (Table 1). Therefore, these variables were unlikely to cause the difference in bullfrog population establishment in water bodies between plots with a simple enclosure and those with an elaborate one. The greater likelihood of bullfrog populations becoming established in water bodies of plots with simple enclosures may be due to more opportunities for bullfrogs to escape from them, which may increase propagule pressures in water bodies of plots with such an enclosure. This is because simple enclosures are more easily destroyed by storms, floods or other factors.

Our findings also revealed several other factors related to the establishment of bullfrog populations. The number of bullfrogs raised on farms was positively related to the establishment of bullfrog populations [41]. Assuming that each individual in a farm has the same chance to escape, increasing the number of bullfrogs raised will promote the number that could escape, resulting in an increase in propagule pressure. There was a negative relationship between hunting pressure and the establishment of feral bullfrogs, which is consistent with results of other studies [41], [42]. This suggests the generality of hunting pressure on the establishment of bullfrog populations. Hunting probably reduces bullfrog survival and the breeding chance of females through higher hunting pressure on males [41].

Bullfrogs inhabit permanent water bodies such as ponds or reservoirs [43]–[45]. The availability of permanent water bodies is critical for bullfrog physiological and ecological requirements at all stages of their life history, and therefore related to their establishment. Ficetola et al. [42] found that human footprint positively related to the likelihood of bullfrog population establishment. In our study, however, human footprint was not retained in the MAM, suggesting that it might had little effect on the establishment of bullfrog populations in this study. The distance from a water body to a bullfrog farm negatively related to the establishment of bullfrogs because it may influence propagule pressures in the distant water bodies. Fewer post-metamorphic bullfrogs can disperse longer distances [48], [49]. They have a reduced chance of dispersal to distant water bodies than closer ones, resulting in a lower chance of the establishment in the distant water bodies.

Ficetola et al. [42] also suggested a positive relationship between the likelihood of the establishment of bullfrog populations and climate. Suitable areas for bullfrogs generally have a minimum temperature ranging between −20°C and +14°C [42]. In this study, none of four climate variables in the Ficetola et al. study [42] were entered into the MAM. This arose because minimum temperatures ranged from −7.1°C to +10.9°C in all plots, and were therefore unlikely to relate to the establishment of bullfrogs. A classic paradigm in invasion ecology is that a greater number of native species would have a higher resistance to biological invasions [50]. The relationship between the likelihood of establishment of bullfrogs in water bodies and native frog species richness was not negative, indicating little resistance of native frogs to bullfrog invasion. A number of studies have documented no effects of species richness in native communities on the establishment of feral populations of introduced animals [42], [51], [52]. Some studies, however, found that non-native coevolved fish could facilitate bullfrog invasion in the United States [39]. We did not find any correlation between the establishment of bullfrogs and the presence of red swamp crayfish or other introduced fish, indicating that these alien species did not relate to the establishment of bullfrogs in the study areas.

The management of biological invasions remains one of the biggest challenges to conservation biologists [5]. Understanding the risks associated with escapes from different aquaculture enclosures can help to decrease the likelihood of population establishment for introduced species, and is critical for providing immediate easy steps that managers and policymakers can undertake. Our study quantified the roles of different aquaculture enclosures in the establishment of breeding populations of bullfrogs in the wild. Because different enclosures can have different effects on the establishment of feral populations of introduced species, improving cultivation enclosures should reduce invasions. The choice of appropriate enclosures is an important measure for managing introduced species. There is a need to improve cultivation technology by using stronger and more durable enclosures to prevent escape and decrease invasion likelihood. In addition to proper enclosures, the appropriate location of farms is also important. They should be far from suitable habitats for introduced species [53].

There have been no regulations on the quality of cultivation enclosures for introduced species in many countries. Our results highlighted the importance of managing enclosure quality and the location of farms, and goverments should draft management regulations. These regulations should standardize cultivation technology for introduced species to reduce invasion risk, and prohibit the use of simple enclosures and inappropriate locations of cultivation farms that would increase the likelihood of introduced species becoming established.

Predicting the potential distribution of invasive species is an effective approach to developing an initial management strategy. Climate suitability was commonly used as the most important factor for predicting the potential distribution of invasive species. Our results have indicated the roles of rearing enclosures on the likelihood of feral population establishment of introduced species, and therefore supplied helpful information for further predictions.

## Materials and Methods

### Study area

Our field samples covered four provinces: Hunan, Hubei, Guizhou and Yunnan (21.14°−33.28° N and 97.53°−116.12° E) (Figure 1), which used to have, or still have, numerous bullfrog farms. These provinces form a continuous area across Central South and Southwest China. Hubei, in the north subtropical monsoon climate zone, is covered with deciduous broad-leaved forest and subtropical evergreen broad-leaved forest, while Hunan with a continental subtropical humid monsoon climate, is covered mainly with subtropical evergreen broad-leaved forest. Guizhou and Yunnan, located in the Yunnan-Guizhou Plateau are topographically and climatically diverse. Guizhou has a subtropical humid monsoon climate with vegetation ranging from subtropical evergreen broad-leaved forest to deciduous broad-leaved forest. Yunnan is in a subtropical humid monsoon climate zone that includes tropical, subtropical, temperate and boreal climates. The vegetation in Yunnan is divided into tropical and monsoon forest, subtropical evergreen broad-leaved forest and subalpine coniferous forest. Four provinces are also rich in water resources. Hunan and Hubei are located in the middle reaches of the Yangtze River, while Guizhou is located in the upper regions and branches of the Yangtze and Pearl Rivers. Yunnan has the Yangtze, Pearl, Yuanjiang, Lancang, Nujiang and Irrawaddy river systems [54]. These complex natural environments result in a rich amphibian diversity in these provinces [54]–[56], particularly in Yunnan and Guizhou.

Many alien species have invaded water bodies in these provinces. For example, red swamp crayfish invaded these areas in the 1930s to 1940s [40]. Some alien fish species such as the Taihu Icefish (*Neosalanx taihuensis*), Grass Carp (*Ctenopharyngodn idellus*), Bighead (*Aristichthys nobilis*), Paradise Stream Goby (*Rhinogobius giurinus*) and Topmouth Gudgeon (*Pseudorasbora parva*), were introduced for aquaculture and other reasons from other Chinese lakes and rivers to water bodies at higher elevations in Yunnan and Guizhou. Other introduced fish species include *Gambusia affinis* from Central and North America, and *Oreochromis spp.* from South Africa [40], [57].

### Methods

We looked for farmers who used to raise or are still raising bullfrogs in the rural areas of four provinces. We sampled more areas in Guizhou and Yunnan Provinces due to their complicated geography. At the time we conducted our study, most farmers had not raised bullfrogs for several years because of poor economic returns. Only farms that were still in agricultural production were used in our study. There are large variations in dispersal distance among bullftogs [48], [49], [58]. Most bullfrog-invaded water bodies in this study were close to bullfrog farms, and bullfrog populations established in water bodies further than 1000 m from bullfrog farms were very rare (less than 2%). We therefore sampled a circular plot with a radius of 1000 m centered at the farm, when one was found. Our analysis excluded those plots that had more than one bullfrog farm in a plot, or where distances between two plots was less than 3 km. This would have made it difficult to determine the most likely source of bullfrogs in the invaded waters in such plots. In each plot we collected data on the types of farm enclosure, the number of bullfrogs raised, the period during which bullfrogs had been raised, the establishment of feral bullfrog populations in water bodies, characteristics and locations of water bodies, the distance of water bodies to the farm, native frog species richness, occurrence of alien red swamp crayfish (*Procambius clarkii*) and fish in water bodies, human activities such as frog hunting activities and human footprint (see below). We surveyed these plots in the bullfrog breeding season between the end of April and the end of July in 2008.

### Number and duration of bullfrogs raised in different enclosures

We designed a questionnaire and interviewed bullfrog farmers who had raised or are still raising bullfrogs [41]. Farmers were asked how many, and over what period, bullfrogs were raised. Most farmers provided information on the area of their farms and the approximate density of bullfrogs within the area. We therefore multiplied the area by bullfrog density and converted this to the total number of bullfrogs raised on the farm. Whenever possible, we also measured the area of the farm, which usually matched the information given to us by the farmers. If there was a discrepancy, our measurement was used. We also recorded the type of farm enclosures. We recorded the location of a farm using a Global Positioning System (eXplorist210, USA). We excluded those samples where farmers were either unwilling or unable to provide us with the required information.

### The establishment of feral bullfrog populations and presence of alien crayfish and fish

We defined the establishment of a feral bullfrog population in a water body in a plot as a wild reproducing population of bullfrogs, identified by the presence of both adults and sub-adults or tadpoles or eggs in the water [41], [59]. We searched for adults, sub-adults, tadpoles and eggs in plots by following transects along all available water courses at a speed of 1.5–2 km/h between 1900–22.30 h at night with an electric torch (12 volt DC lamp) [41]. We also listened carefully for bullfrog calls. At the same time, we recorded if crayfish were present in each water body. The Aquatic Culture Departments of local governments generally are responsible for monitoring or controlling alien fish introduction into local ponds, pools and reservoirs. We obtained data on records of alien fish introduction into each water body by visiting Aquatic Culture Departments. We recorded the establishment of a feral bullfrog population and the occurrence of alien crayfish and fish in a water body as a dichotomous variable with establishment/presence as 1 and failure/absence as 0.

### Native frog richness and characteristics in water bodies

When surveying bullfrog populations, we also investigated the species richness of native frogs, and the characteristics of the water bodies in a plot, including surface area, maximum depth, submerged vegetation cover, shade and the presence of permanent water bodies. The location of the farm enclosure and each water body in a plot was recorded using the GPS. Line transect methods were used to investigate species richness of native frogs in the water bodies [60]. Transects (2 m×10 m) followed the shoreline with half of the width of the transect (1 m) in the water and half on the shore. The accessible shoreline of each water body was divided into segments of equal length, excluding parts constructed from stones or cement. For waters <200 m^2^ in area, the shoreline was divided into 2–4 segments, and for those >200 m^2^ into five segments. One line transect was located at random within each segment. Each water body was surveyed for three consecutive nights. Transects were located in a different randomly chosen position each night. Frog species were identified by sight with the help of guidebooks [61].

For reservoirs or ponds (most water bodies), maximum depth was estimated as the difference in height between the water surface and the bottom of the dam. For rice fields, pools or rivers, which are shallow and usually less than 3 m in depth, maximum depth was measured in an accessible area. The surface area of a body of water in the plot was estimated according to its geometrical shape. Following the frog line transect survey (see above), the cover of submerged vegetation in a 1 m wide strip from the water's edge in the water part of each transect was estimated and assigned to one of 11 categories: 1 (0%), 2(1%–10%), 3 (11–20%),…9 (71–80%), 10 (81–90%), and 11(>90%) [41]. We estimated shade in a water body by measuring the angle from the water body center at eye level (165 cm in height) to the top of the tree line or horizon east, south and west using a handheld clinometer [62]. We used the average of angles as an index of shade. A water body was defined as permanent water if it was not dry at the end of the dry season [39]. We also recorded the presence or absence of permanent water bodies in a plot as a dichotomous variable with presence as 1 and absence as 0.

### Frog hunting pressure

We designed a questionnaire for the hunting pressure survey based on that of Li et al. [27]. We asked two local farmers in each village with bullfrog farms whether there was frog hunting activity at night. Of the two farmers, one was a bullfrog farmer whilst the other was a crop farmer. Both were concerned about hunting activities on their farms because hunters can damage their bullfrogs or crops. The interviewees always gave us one of two answers, either “no hunting or occasional hunting” or “frequent hunting.” The results of questionnaire surveys from two interviewers in a village were highly consistent. We therefore classified the data as a dichotomous variable as follows: 0, no hunting or occasional hunting; 1, frequent hunting.

### Human footprint and climate variables

We collected data on human footprint and climate variables from available GIS data, following the study of Ficetola et al. [42]. The human footprint refers to a composite factor, integrating population density and human modifications [63], which may facilitate bullfrog invasion [42], [46], [47]. We selected four climate variables [42]: Tmax (maximum temperature of the hottest month), Tmin (minimum temperature of the coldest month), Prec1202 (summed precipitation between December and February) and Prec0608 (summed precipitation between June and August). These four variables were considered to have avoided the multicollinearity issues and described the need of bullfrogs for thermal energy and water availability [42]. We obtained the human footprint data from the last-of-the-wild web site (http://www.ciesin.columbia.edu/wild_areas/), and the climate variables from the WorldClim database with a spatial resolution of 30 seconds from 1950 to 2000 [64] (available at http://www.worldclim.org).

### Statistical Analysis

The area of a water body and the number of bullfrogs raised in farms were log_10_ transformed to improve normality for analysis. We first compared differences in the likelihood of establishment of feral bullfrog populations, characteristics of farms and water bodies, the occurrence of red swamp crayfish and alien fish, and the occurrence of frequent hunting and human footprint in water bodies between plots with a simple enclosure and those with an elaborate enclosure using t-tests for continuous variables and χ^2^ tests for percentage variable. We then used a generalized linear mixed model with a binomial error structure and a logit link function to derive the relationships between establishment or failure of a bullfrog population as dependent variables and factors that potentially related to the establishment or failure as independent variables. This model accounted for the possibility of spatial pseudoreplication of several water bodies in a plot by assigning plot identity as a random variable. Due to multicollinearity among predictors, we selected the factors for the model as follows. Eight variables including number of bullfrogs raised, occurrence of frequent hunting, human footprint, the occurrence of permanent water, Tmax, Tmin, Prec1202 and Prec0608 were selected a priori as potentially important for the establishment of bullfrog populations based on published research [41], [42], [45]–[47], [49], [65]–[67]. We selected other predictors by running a generalized linear mixed model with a single variable, which significantly related to the establishment of bullfrog populations. We then fitted the generalized linear mixed models with the selected factors as predictors. We determined a minimum adequate model (MAM) using the Akaike Information Criterion (AIC) [68], [69], after fitting the full model. We used backward stepwise selection to reduce a full model containing all independent variables to the model with the lowest AIC value, by sequential deletion of the variable with the lowest contribution to the model at each step. We checked that the addition of no variable to this MAM further lowered its AIC. All analyses were conducted with R version 2.8.1 (R Development Core Team, 2008). We performed linear mixed models using S4 classes version 0.999375-28 in R. The data we used are included in the Appendix S1.

## Supporting Information

Appendix S1 Data on 20 predictor variables and establishment or failure of a bullfrog population in 137 water bodies
(0.06 MB XLS)
